# Changes of Maximum Leg Strength Indices During Adulthood a Cross-Sectional Study With Non-athletic Men Aged 19–91

**DOI:** 10.3389/fphys.2018.01524

**Published:** 2018-11-01

**Authors:** Wolfgang Kemmler, Simon von Stengel, Daniel Schoene, Matthias Kohl

**Affiliations:** ^1^Institute of Medical Physics, University of Erlangen-Nürnberg, Erlangen, Germany; ^2^Faculty of Medical and Life Sciences, University of Furtwangen, Schwenningen, Germany

**Keywords:** maximum lower extremity strength, sarcopenia, dynamopenia, strength decline, leg-press

## Abstract

Age-related loss of muscle mass and function, also called sarcopenia, was recently added to the ICD-10 as an independent condition. However, declines in muscle mass and function are inevitable during the adulthood aging process. Concerning muscle strength as a crucial aspect of muscle function, maximum knee extension strength might be the most important physical parameter for independent living in the community. In this study, we aimed to determine the age-related decline in maximum isokinetic knee extension (MIES) and flexion strength (MIFS) in adult men. The primary study hypothesis was that there is a slight gradual decrease of MIES up to ≈age 60 years with a significant acceleration of decline after this “changepoint.” We used a closed kinetic chain system (leg-press), which is seen as providing functionally more relevant results on maximum strength, to determine changes in maximum isokinetic hip/leg extensor (MIES) and flexor strength (MIFS) during adulthood in men. Apart from average annual changes, we aimed to identify whether the decline in maximum lower extremity strength is linear. MIES and MIFS data determined by an isokinetic leg-press of 362 non-athletic, healthy, and community-dwelling men 19–91 years old were included in the analysis. A changepoint analysis was conducted based on a multiple regression analysis adjusted for selected co-variables that might confound the proper relationship between age and maximum strength. In summary, maximum isokinetic leg-strength decline during adulthood averaged around 0.8–1.0% p.a.; however, the reduction was far from linear. MIES demonstrated a non-significant reduction of 5.2 N/p.a. (≈0.15% p.a.) up to the estimated breakpoint of 52.0 years and an accelerated loss of 44.0 N/p.a. (≈1.3% p.a.; *p* < 0.001). In parallel, the decline in MIFS (10.0 N/p.a.; ≈0.5% p.a.) prior to the breakpoint at age 59.0 years was significantly more pronounced. Nevertheless, we observed a further marked accelerated loss of MIFS (25.0 N/p.a.; ≈1.3% p.a.) in men ≥60 years. Apart from the “normative value” and closed kinetic chain aspect of this study, the practical application of our results suggests that sarcopenia prophylaxis in men should be started in the 5th decade in order to address the accelerated muscle decline of advanced age.

## Introduction

Sarcopenia, characterized as a reduction of muscular mass and -function (Cruz-Jentoft et al., 2010; Fielding et al., 2011; Studenski et al., 2014) was included in the ICD-10 CM^1^ code as a musculoskeletal disease in 2016 (M62.84). Although the relevance of muscle mass for healthy aging might be underestimated^2^; functional or more dedicated “dynamopenic”^3^ (Greco et al., 2014) aspects are without doubt more important for older people's well-being and independent living.

In this context, studies have reported the particular crucial relevance of age-dependent declines in leg-extension/quadriceps strength on mobility limitations, disability, morbidity, and mortality in older people (Visser et al., 2005; Newman et al., 2006; Roshanravan et al., 2017). Unfortunately, the reduction in muscle strength in older age was reported to be much more pronounced (Goodpaster et al., 2006; Dey et al., 2009; Koster et al., 2011) than the decline in muscle mass. Further, maximum strength deterioration of the lower limbs was much higher compared with upper limbs (Viitasalo et al., 1985; Frontera et al., 2000; Landers et al., 2001; Dey et al., 2009; Amaral et al., 2014).

However, age-related declines in muscle mass, strength and function are inevitable developments in human adults. Nevertheless, what is the “normal age-appropriate” decline of muscle mass and function? Further, is this decline linear over the adult lifespan or are there changepoints of an accelerated loss of lower extremity muscle parameters?

Considering the essential effect of sex steroids GH, and IGF-I in muscle protein synthesis, the rapid menopausal reduction of both estrogens/testosterone (Veldhuis, 2008; Decaroli and Rochira, 2017) and GH/IGF-I (Sherlock and Toogood, 2007) suggests evidence for an accelerated decline of muscle mass and strength during women's early postmenopausal years (Maltais et al., 2009). However, (bioavailable) testosterone and corresponding declines in GH/IGF-I in men are much more linear (Harman et al., 2001; Veldhuis, 2008; Veldhuis et al., 2009; Decaroli and Rochira, 2017). Further, declines of muscle mass predominately affected by low serum concentration of anabolic agents did not consistently correlate with corresponding strength changes (Kim et al., 2018). Nevertheless the few data that focus on this issue (e.g., Larsson et al., 1979; Frontera et al., 1991; Lindle et al., 1997; Akima et al., 2001; Harbo et al., 2012) point to periods of accelerated strength decline of lower leg strength indices also in men.

However, there is no consensus as to when accelerated muscle strength decline starts. While most researchers (Larsson et al., 1979; Frontera et al., 1991; Akima et al., 2001; Harbo et al., 2012) located the accelerated decline in the mid and late 50ies, one author applied a more sophisticated statistical approach (Lindle et al., 1997) that led him to suggest a much earlier start of accelerated strength loss (40ies).

One may argue that assessing the amount and time pattern of lower extremity strength declines in adults is a somewhat academic exercise. We do not accept that idea because in actual fact normative data of strength changes and potential accelerated strength decline changepoints form the basis for clinical decisions and corresponding therapeutic approaches. This might in particular be the springboard for more reliable and sophisticated strength testing which otherwise suffer from a lack of normative data.

The aim of the present contribution was thus to determine the decline in maximum isokinetic hip/leg extensor (MIES) and flexor strength (MIFS) as determined by an isokinetic leg press during adulthood. Based on the research question discussed above we focus on a male cohort 19–91 years old. Our primary hypothesis was that there is a linear decrease of MIES up to ≈age 60 years with a significant acceleration of decline after this “changepoint.” Our secondary hypothesis was that there is a linear decrease of MIFS up to ≈age 60 years with a significant acceleration of decline after this “changepoint.” Lastly, an experimental hypothesis was that adjusted for lean body mass (LBM) both MIES and MIFS loss their “significant changepoint.”

## Methods

**Figure 1:**
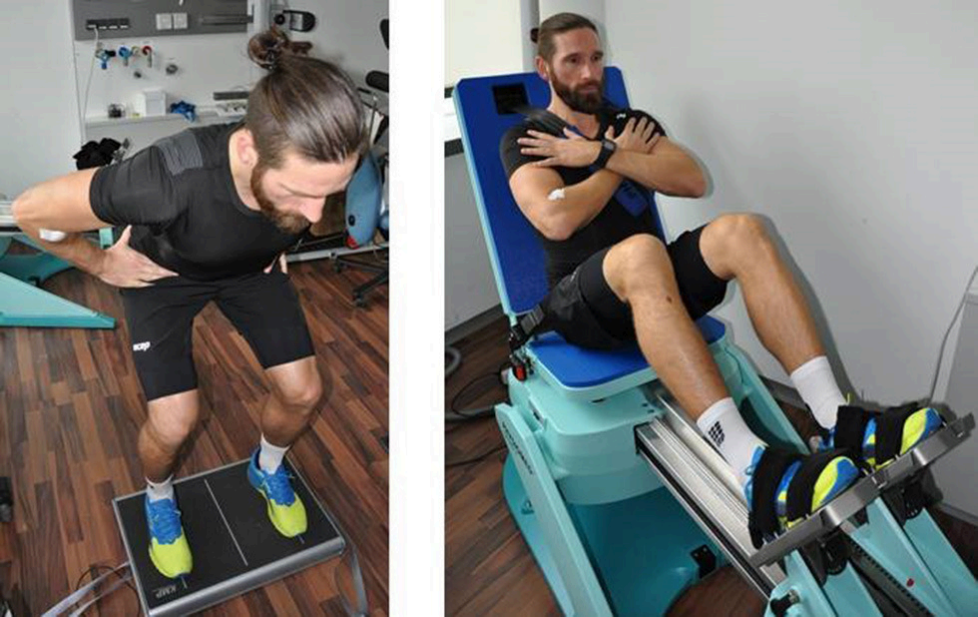
Leg press test (hip/leg extensor, hip/leg flexor strength) conducted on an isokinetic device (CON-TREX LP, Physiomed, Laipersdorf, Germany).

We used isokinetic leg-press data from several previous and ongoing cross-sectional and longitudinal^4^ projects (e.g., Kemmler et al., 2010, 2014, 2016a, 2017) with men of different ages to evaluate the present research issue. All the studies were conducted between February 2008 and May 2018 by the Institute of Medical Physics (IMP), Friedrich-Alexander University (FAU) of Erlangen-Nürnberg. All the projects were approved by the university ethics committee of the FAU, Germany and fully complied with the Helsinki Declaration “Ethical Principles for Medical Research Involving Human Subjects.” After detailed information, all the study participants gave their written informed consent. This also refer to the person in Figure 1, who provide written informed consent for his image to be published.

### Outcomes

Primary study-endpoint

- Maximum dynamic strength (peak torque) of the leg/hip extensors (MIES).

Secondary study-endpoints

- Maximum dynamic strength (peak torque) of the leg/hip flexors (MIFS).

### Participants

Altogether 362 community dwelling men 19–91 years old were included in the present analysis. Inclusion criteria were (1) male gender, (2) legal age, (3) community dwelling (cdw), i.e., independently living in the community. Subject were excluded when (1) taking medication (e.g., glucocorticoids >5 mg/d) or suffering from diseases with relevant impact on muscle metabolism, (2) conducting intense athletic performance (≥3 sessions/week/year), (3) diagnosed as having sarcopenia according to EWGSOP (Cruz-Jentoft et al., 2010), and (4) demonstrating low test compliance or unable to properly perform the isokinetic strength test (Table 1).

**Table 1:** Participant characteristics of 362 community dwelling men 19–91 years old structured in ranges of 15 years.

**Variable**	**<35 yrs**	**35–49 yrs**	**50–64 yrs**	**65–79 yrs**	**80 yrs+**
*N*	70	121	51	75	29
Age [years]	27.4 ± 5.2	43.6 ± 4.0	55.6 ± 4.7	71.4 ± 3.2	83.6 ± 3.0
Body height [cm]	182.5 ± 7.7	180.3 ± 7.0	180.8 ± 5.6	171.0 ± 5.6	170.0 ± 5.9
Body mass [kg]	82.4 ± 11.8	87.8 ± 13.8	88.4 ± 13.2	78.4 ± 10.1	74.5 ± 9.1
BMI [kg/m^2^]	24.7 ± 3.7	27.0 ± 3.9	27.0 ± 3.6	26.8 ± 2.7	25.8 ± 2.5
Lean body mass [kg]	66.9 ± 8.9	67.5 ± 8.3	65.9 ± 8.3	58.8 ± 6.9	56.6 ± 4.7
SMI [kg/m^2^]	8.68 ± 0.83	8.70 ± 0.90	8.52 ± 0.77	8.13 ± 0.70	7.96 ± 0.61
Body fat [%]	18.8 ± 4.1	23.1 ± 5.0	25.4 ± 4.9	25.0 ± 4.6	24.0 ± 4.8
Smokers [%/group]	13%	9%	10%	7%	7%
Activity Index^a^	3.3 ± 1.2	3.2 ± 1.3	3.4 ± 1.5	4.0 ± 1.3	3.9 ± 1.5
Exercise [min/week]	71 ± 48	51 ± 41	42 ± 37	35 ± 38	27 ± 36
MIES [N]	3,553 ± 584	3,373 ± 595	3,144 ± 621	2,141 ± 553	1,767 ± 425
MIES/LBM [N/kg]	53.2 ± 10.5	49.9 ± 9.0	47.7 ± 8.9	36.4 ± 8.7	31.2 ± 7.5
MIFS [N]	1,911 ± 372	1,593 ±325	1,506 ± 358	948 ± 270	729 ± 177
MIFS/LBM [N/kg]	28.6 ± 6.2	23.6 ± 5.0	22.9 ± 5.5	16.1 ± 4.2	12.9 ± 3.5
MIFS/MIES-Index	0.538 ± 0.080	0.472 ± 0.083	0.479 ± 0.102	0.443 ± 0.089	0.413 ± 0.70

*BMI, Body Mass Index; SMI, Skeletal muscle mass index according to Baumgartner et al. (1998); i.e., appendicular skeletal muscle mass/body height2. MIES, maximum isokinetic hip/leg extensor strength; MIFS, maximum isokinetic hip/leg flexor strength*.

a *Scale from (1) very low to (7) very high (Kemmler et al., 2004)*.

### Assessments

Body-height was measured by a Harpender stadiometer (Holtain, Crosswell, UK); body mass and composition was determined via direct-segmental, multi-frequency Bio-Impedance-Analysis (DSM-BIA; InBody 230/770, Seoul, Korea) applying standardized protocols. BMI was calculated body mass/square body height; skeletal muscle mass index (SMI) was calculated according to Baumgartner et al. (1998) (i.e., appendicular skeletal muscle mass/square body height). Lean body mass was defined as fat-free body mass. Body fat as listed in Table 1 refers to the fat rate for the total body. In order to standardize the BIA assessment, we consistently use the same BIA test protocol that includes minor physical activity for 8 h and 15 min of rest in a supine position immediately before the BIA assessment. Further, all participants were provided with written specifications about dos and don'ts including basic nutritional guidance 24 before testing. Baseline characteristics including physical activity and exercise were determined by questionnaires. For details, the reader is kindly referred to another publication (Kemmler et al., 2004).

Maximum isokinetic strength of the leg and hip extensors/flexors was tested using an isokinetic leg press (CON-TREX LP, Physiomed, Laipersdorf, Germany) (Figure 1). Bilateral hip/leg extension and flexion was performed in a sitting, slightly supine position (15°), supported by hip and chest straps. Range of motion was selected between 30 to 90° of the knee angle, with the ankle flexed 90° and feet firmly fixed with straps positioned on a flexible sliding footplate. The standard default setting of 0.5 m/s was used. We consistently used our standard test specifications for all cohorts. Starting with a 5 min warm up on a cycling ergometer, one familiarization trial (5 repetitions) with the dedicated movement pattern (“push and pull”), and 3 min of rest, participants were then asked to conduct five repetitions with maximum voluntary effort. Participants conducted two trials intermitted by 2 min of rest. We consistently included the highest value for hip/leg extension and hip/leg flexion of the five repetitions and both trials in the data analysis. Hip/leg flexor (MIFS)/hip/leg extensor isokinetic strength (MIES)-Index (MIFS/MIES-Index) was calculated as MIFS divided by MIES. Reliability for the maximum hip/leg extensor strength (Test-Retest-Reliability; Intra Class Correlation) was 0.88 (95%-CI: 0.82–0.93) for a male cohort 30–50 years old. The same test assessor responsible for the leg-press assessments conducted most of the tests.

### Statistical analysis

Based on a statistically (Shapiro-Wilk test) and graphically (Q-Q plots) checked normal distribution, the outcomes presented in Table 1 were reported using mean values (MV) and standard deviation (SD). We abstained from a sophisticated statistical procedure for participant characterics structured according to age (Table 1). However, we statistically addressed the issue of overall strength changes during adulthood for MIES and MIFS comparing the oldest with the youngest subgroup using pairwise (independent) *t*-tests and Cohen's d effect sizes. Although not necessarily specified for subordinate study endpoints, *t*-tests were adjusted for multiple testing using the Bonferroni procedure. The statistical procedures listed above were performed using SPSS Statistics version 25.

Furthermore, we performed a multiple regression analysis with change point estimation using the statistical software R in combination with package segmented (Muggeo, 2008). Dependent variables within the multiple regression were MIES and MIFS while age, body height, body mass, ASMM^5^, and exercise (Table 1) served as independent variables. The significance of the change points was assessed by the score-test proposed by Muggeo (Muggeo, 2016) and additionally verified by Davies' test (Davies, 2002). All tests were 2-tailed, significance was accepted at p < 0.05.

## Results

Table 1 gives the characteristics of the 362 participants 19–91 years old included in the analysis. As evident from Table 1, the age groups were not equally distributed (*p* < 0.001); the lowest sample size was generated for men 80 years+ (*n* = 29). However, more crucially, there was a relatively small number of study participants (*n* = 47) between 51 and 69 years^6^.

Further, Table 1 gives strength indices determined for different age groups. In summary, maximum isokinetic strength of the hip/leg extension (MIES) and hip/leg flexion (MIFS) strength as determined by a closed kinetic system at least halved (MIES: −50 ± 21%, MIFS: −62 ±2 4%) during <35 to ≥80 years (adjusted *p* ≤ 0.001; ES, d': 3.50 and 2.95, respectively).

Adjusting MIES for LBM (Table 1) or BMI (<35 years: 143.9 ± 30.3 vs. ≥80 years: 68.4 ± 17.6 N/[kg/m^2^]) resulted in comparable steep declines (MIES/LBM: 41 ± 15%, ES d' = 2.42; MIES/BMI: 52 ± 22%; ES d' = 3.05).

Correspondingly, MIFS adjusted for BMI decreased from 77.4 ± 17.3 N/[kg/m^2^] at age <35 years to 28.3 ± 7.8 N/[kg/m^2^] at age ≥80 years; i.e., by −63 ± 25% (ES d' = 3.66). In parallel MIFS/LBM (Table 1) decreased by 55±24% (ES d' = 3.12).

Due to the more pronounced decline of MIFS vs. MIES the corresponding index also declined over the adults' age span (adjusted *p* < 0.011; ES, d' = 1.66).

**Figure 2:**
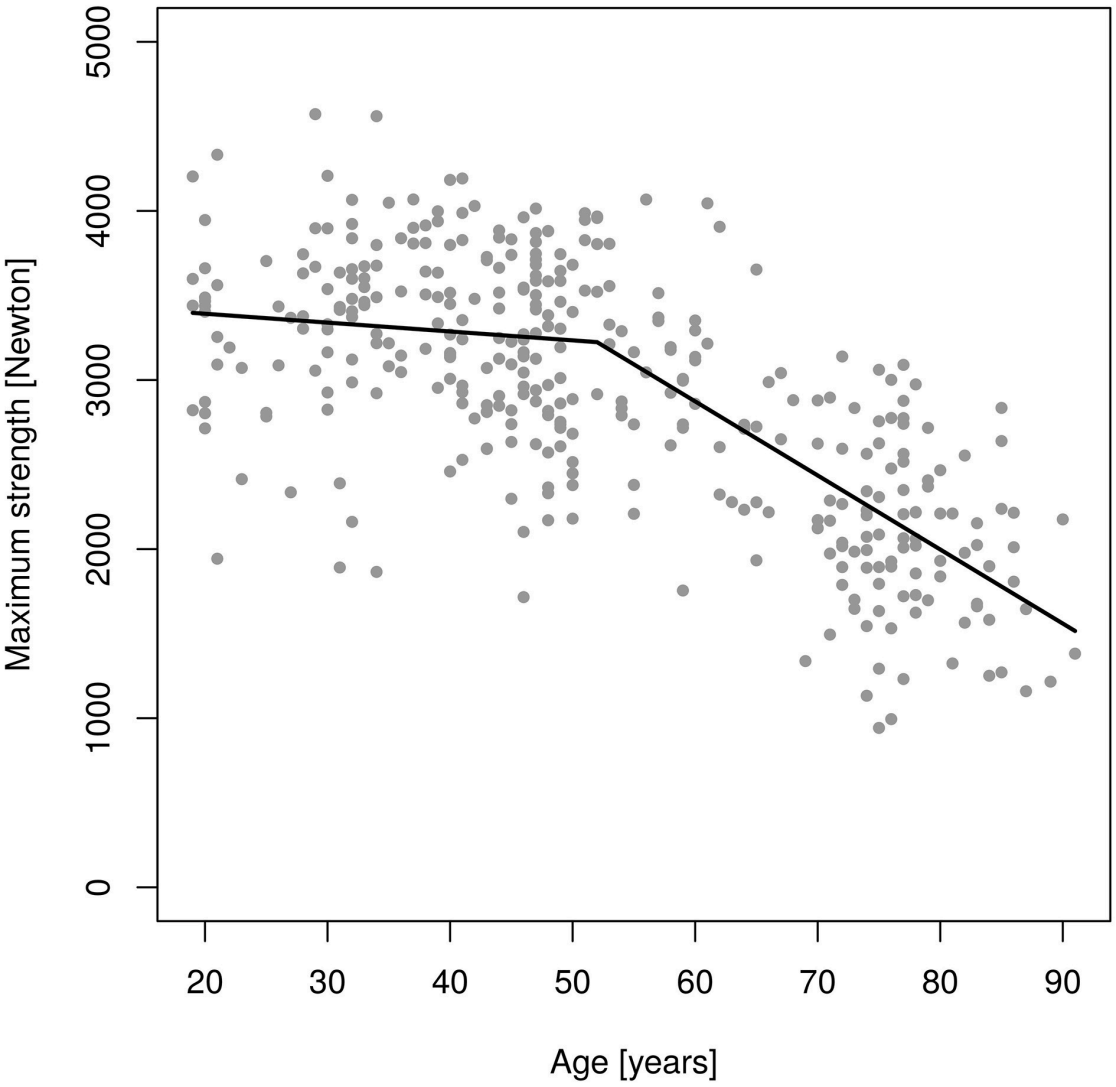
Changes of maximum isokinetic hip/leg extensor strength (MIES) during adulthood as determined by an isokinetic leg-press.

**Figure 3:**
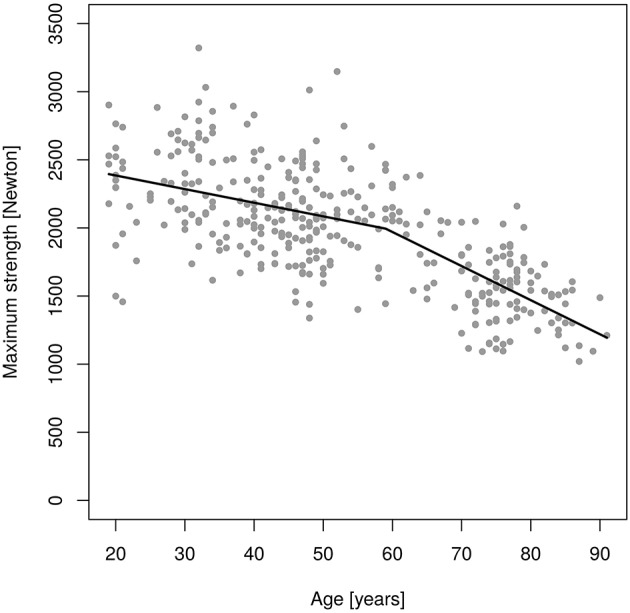
Changes of maximum isokinetic hip/leg flexor strength (MIFS) during adulthood as determined by an isokinetic leg-press.

Figures 2, 3 shows results of the “changepoint analysis” for MIES (Figure 2) and MIFS (Figure 3) after multiple regression analysis adjusted for the co-variables “age,” “body-height,” “body-mass,” “ASMM”^7^ and “exercise.” The latter model explained 61% each of the variance of MIES (*r*^2^: 0.611) and MIFS (*r*^2^: 0.605). In summary, MIES demonstrates a non-significant reduction of 5.2 N/p.a. (95% CI: 3.2 to −13.7 N/p.a.; i.e., ≈0.15% p.a.) up to the estimated breakpoint of 52.0 years (*p* < 0.001) and an accelerated loss of ≈44 N/p.a. (95% CI: −34.5 to −53.1 N/p.a.; i.e., ≈1.3% p.a.) after this changepoint. Of importance, the more conservative Davies-Test (Davies, 2002) confirmed (*p* < 0.001) the significant changepoint^8^ at age 52 years; a second significant changepoint was not determined. Thus, we have to revise our primary hypothesis that there is a linear decrease of MIES up to age ≈60 years with a significant acceleration of decline after this “changepoint.”

In parallel to MIES, only one significant changepoint (*p* = 0.001) was determined for MIFS (Figure 3). However, in contrast to MIES the decline of MIFS prior to the breakpoint at age 59.0 years was already significant. Correspondingly, younger men lost 10.0 N/p.a. (−5.8 to −14.2 N/p.a.; i.e., ≈0.5% p.a.), while the decline in MIFS in men 60 years and older averaged 25.0 N/p.a. (−6.2 to −32.9 N/p.a.; i.e., ≈1.3% p.a.). The Davies-Test confirmed (*p* < 0.004) the significant changepoint at age 59 years. Thus, we confirmed our hypothesis that there is a linear decrease of MIES up to ≈age 60 years with a significant acceleration of decline after this “changepoint.” Correspondingly, we confirmed our secondary hypothesis that there is a linear decrease of MIES up to ≈age 60 years with a significant acceleration of decline after this “changepoint.”

Applying the same statistical procedures for MIES and MIFS, we are unable to observe changepoints for a significant decline (*p* > 0.100) after adjusting for lean body mass (i.e., N/kg LBM) for either MIES or MIFS. Thus, we confirmed our experimental hypothesis I that adjusted for LBM both MIES and MIFS lost their “significant changepoint.”

## Discussion

In this article, we particularly aimed to determine the amount and progress of age-dependent reductions of maximum lower limb strength in community-dwelling men. Our most striking motivation for focusing on hip/leg extension (and flexion) strength was the particular relevance of leg-extension strength on mobility limitations, disability, morbidity, and mortality in older people (Visser et al., 2005; Newman et al., 2006; Roshanravan et al., 2017). Of importance, the present study is the first to provide data on maximum hip/leg extensor and flexor strength over a wide range of male adulthood (19–91 years) using an isokinetic leg press. We consider the approach of applying a closed chain kinetic system important, since these tests might determine functional aspects of lower limb performance in more depth (Augustsson and Thomeé, 2000).

In summary, we determined an average yearly reduction of ≈0.8% p.a. for MIES and ≈1.0% p.a. for MIFS. However, we observed a significant acceleration of decline in MIES that started earlier (52 years) than expected and varied from the later significant decline in MIFS (59 years).

Although we have to admit that it might be inadequate to compare our results with open isokinetic chain testing (Ferraresi et al., 2013), we generally confirmed the course and volume of declines of knee extensor and flexor strength during male adulthood (e.g., Larsson et al., 1979; Frontera et al., 1991; Poulin et al., 1992; Porter et al., 1995; Lindle et al., 1997; Neder et al., 1999; Akima et al., 2001; Harbo et al., 2012; Cheng et al., 2014).

In detail, annual changes in concentric MIES/MIFS reported by studies that assessed men between the age of 20 and 70–80 (e.g., Larsson et al., 1979; Poulin et al., 1992; Porter et al., 1995; Lindle et al., 1997; Neder et al., 1999; Akima et al., 2001; Harbo et al., 2012) averaged between 0.5% p.a. (Lindle et al., 1997) and 1.2% p.a. (Akima et al., 2001). Closest to our results, Akima et al. (2001) reported age-related declines in maximum isokinetic strength of 1.2% p.a. for MIES and 1.1% p.a. for MIFS for their cohort of 100 Japanese men 20–84 years old.

Although it is difficult to compare cross-sectional with longitudinal study results, it might be worthwhile to briefly address age-related changes of lower extremity muscle strength in older people (e.g., Frontera et al., 2000; Goodpaster et al., 2006; Lauretani et al., 2008; Dey et al., 2009; Koster et al., 2011; Roshanravan et al., 2017). In summary, with few exceptions (i.e., Lauretani et al., 2008)^9^ average maximum strength reductions range around 3.0–3.5% p.a. (Goodpaster et al., 2006; Koster et al., 2011) for concentric KES and from 3.3% p.a. (Hicks et al., 2012) up to 9% p.a. for isometric KES (Dey et al., 2009). Corresponding maximum strength declines at advanced age observed in the present study were lower (MIES: 2.0–2.5% p.a.). However, apart from the cross-sectional study design and differences in strength assessment (i.e., open / closed kinetic chain), the most striking difference was that we focus exclusively on community dwelling (CDW) men. This approach might constitute a relevant bias, since people with very low MIES might be unable to “live independently in the community.” Thus, we might have excluded a cohort with (very) low maximum strength and consequently gained lower declines in MIES at older age. However, due to the lack of information, we are not convinced whether all the longitudinal studies account for this aspect.

Revisiting changepoints for accelerated strength loss of the lower extremities, we confirmed the results of Akima et al. (2001), Harbo et al. (2012) and Larsson et al. (1979). Although none of these researchers conducted a dedicated changepoint analysis, they found that the most pronounced decline of isokinetic and isometric knee extensor strength may well-occur in the mid to end 50ies. However, using regression analysis, Lindle et al. (1997) reported an earlier (fourth decade) start of accelerated decline in concentric or eccentric peak torque of the knee extensors in men and women.

Addressing the later changepoint (52 vs. 59 yrs.) for accelerated strength loss in MIES vs. MIFS, we are unable to provide a meaningful physiologic explanation for this feature. For want of corresponding data in the literature, we can only hazard as an explanation that the later decline of MIFS compared with MIES might be related to the more pronounced decline before and less pronounced decline after the MIFS changepoint.

We observed largely parallel changes of LBM and MIES/MIFS (Table 1). Thus, muscle strength declines can be attributed to a considerable extent to declines in muscle mass parameters (Akima et al., 2001), although this relationship might differ between races, at least in men (Araujo et al., 2010). However, relative changes of muscle mass parameters were much lesser pronounced (i.e., LBM: 15 ± 13%) compared with strength changes (MIES: −50 ± 21%, MIFS: −62 ± 24%) an aspect that might explain that adjusting MIES/MIFS for LBM resulted in a more linear decrease of strength declines and a loss of the significant changepoints for MIES and MIFS. However, the high relevance of muscle mass parameters for functional outcomes (here: “strength”) in older individuals is also confirmed by the study of Akima et al. (2001) which concluded that muscle mass dimensions (here: CSA quadriceps femoris) are the “primary factor” involved in an aged individual's capacity to exert maximal force (isokinetic knee flexion and extension). Apart from this aspect, other factors not addressed in our project, e.g., nervous control, muscle recruitment, qualitative changes in contractile properties, etc. (review in Tieland et al., 2018) of course contribute relevantly to age-related changes of muscle strength.

In summary, using a closed kinetic chain testing procedure (i.e., isokinetic leg-press) we largely confirmed the average percentage loss of lower extremity muscle strength during adulthood. Further, and specifically addressed by our main hypothesis, we are in accord with most other studies that the changepoint of accelerated loss of hip/knee extensor and flexor strength is located between ages 50 and 60 years. So far, differences between open and closed kinetic chain-based testing with corresponding impact of functional performance were not remarkable.

However, our study features some particularities and limitations that may prevent a proper interpretation of our results and an adequate comparison with other studies in this field. (1) One may dispute the practical relevance of isokinetic testing in the context of sarcopenia/dynamopenia. While isokinetic movements may not represent a challenge in daily living, functional tests of the lower legs (i.e., squats, lunges) are more akin to daily activities. Reviewing the literature, correlations between isokinetic and functional testing vary from moderate (Augustsson and Thomeé, 2000) to high (e.g., Butcher et al., 2012; Gkrilias et al., 2018); the results do, however, depend on the specifications of the tests (e.g., °/s) and the cohorts tested. Further, most results were reported for isokinetic chairs, with their leg extension movement that differ considerably from the functionally more relevant closed kinetic chain exercise “leg-press.” However, we finally opted to use an isokinetic test device for this research issue because of the higher degree of standardization that it offers. (2) Age groups were not equally populated and there is a relative lack of participants between 51 and 69 years old (*n* = 47), i.e., in the range of the expected changepoints of accelerated strength decline. (3) Although age-dependent BMI was inconspicuous and comparable to normative data for German men (Statistisches Bundesamt, 2012), there is a highly significant decline of body height between 50–64 and 65 years+. (4) Body fat rate of all the age groups is considerably lower (i.e., %-LBM was higher) than reported for German cohorts of comparable age and physical activity. Particularly in our youngest group, the body fat was quite low for a non-athletic population. However, based on our eligibility criteria (healthy, non-athletic, and cdw men, without medication known to affect muscle metabolism), in summary we consider our cohort as representative for the majority of the basic male population. Accordingly, we decided that the results of MIES, MIFS and MIFS/MIES-Index listed in Table 1 can be widely considered as “normative data” for the corresponding age groups. (5) Most, but not all tests were consistently conducted by the same test assessor; further, we consistently applied our standardized test protocol across all studies. (6) Finally, we focus on cdw men, i.e., predominately men with a functional status that permits independent living in the community. Potentially, this might have generated a relevant bias, since institutionalized and correspondingly more functionally limited older men did not contribute to the result of our study. This estimation is confirmed by the rather high MIES and MIFS in our oldest cohort.

A limited amount of studies clearly focus on the issue of age-dependent strength declines. The present study adds further evidence that lower extremity strength decreases considerably during adult men's lives; however the more salient result was that this decline is far from linear. Summarizing the novel aspects and practical application of our study: (1) For the first time “normative values” of maximum isokinetic lower extremity strength have been provided for a closed isokinetic chain system. (2) Further, this is the first study to apply a sophisticated statistical approach to determine the precise changepoints for accelerated muscle loss for MIES and MIFS (3) Unlike most studies that focus on a narrow age range, the present study covers the full range of adulthood. (4) From a practical application point of view, our results provided further evidence that programs that focus on sarcopenia prophylaxis (…or at least its dynamopenic aspect) in men should be started in the 5th decade. Of importance, a related study that focused on trainability in different periods of life (Von Stengel and Kemmler, 2018)^10^ reported a significant trainability of MIES and MIFS in all the age groups addressed here. This included our oldest cohort of men 80 years+, an observation confirmed by various longitudinal studies (review in Stewart et al., 2014).

In conclusion, due to the accelerated loss of muscle strength starting in the 50ies, health care programs that focus not only but specifically on the “dynamopenic” aspect of sarcopenia prevention should start early in men's lives. We speculate that due to the occupational and social situation of male subjects aged about 50 years characterized, inter alia, by a general lack of time^11^, there might be a need for dedicated exercise programs for this cohort. Time-effective exercise protocols [i.e., HIT-single set resistance exercise and/or whole-body electromyostimulation (Kemmler et al., 2016a,b)], ideally combined with adjuvant nutritional support (e.g. protein, BCAA), i.e., strategies recognized to increase muscle mass and strength (Tipton, 2008; Cermak et al., 2012) might be thus a feasible option for this cohort.

## Data availability

The anonymized data used to support the findings of this study are available from the corresponding author upon request.

## Author contributions

SvS, DS, MK, and WK designed the study, completed data analysis and/or interpretation and drafted the manuscript. SvS, MK, DS, and WK contributed to study conception and design and revised the manuscript. WK accepts full responsibility for the integrity of the data sampling, analysis and interpretation.

### Conflict of interest statement

The authors declare that the research was conducted in the absence of any commercial or financial relationships that could be construed as a potential conflict of interest.

## Footnotes

^1^ICD: International Classification of Diseases and Related Health Problems; CM: Clinical Modification.

^2^….considering its relevance for resting metabolic rate and thermoregulation (Kenney and Buskirk, 1995; Kim et al., 2014).

^3^Greek origin: “dynamae” (δ$\overset{\cdot }{\text{u}}$ναμη), c.f. dyna*mo*meter not dynameter.

^4^We used only the baseline data of these studies.

^5^Appendicular skeletal muscle mass i.e. muscle mass of the limbs. ASMM is the crucial muscle mass parameter within the sarcopenia criteria.

^6^i.e. in the range of our expected changes points.

^7^Appendicular skeletal muscle mass.

^8^i.e., the point at which the significantly accelerated decline started.

^9^However, the 6-year follow-up of the study (Lauretani et al., 2008) reported only an overall 0.8 kg (5.1%) decline in isometric KES.

^10^The authors analyzed the longitudinal results on MIES and MIFS of studies that were also included in the present study (…however, baseline data only).

^11^….the reason predominately cited for not exercising (Rütten et al., 2009).
